# Enhanced Electrocatalytic Activity and Stability toward the Oxygen Reduction Reaction with Unprotected Pt Nanoclusters

**DOI:** 10.3390/nano8110955

**Published:** 2018-11-20

**Authors:** Jing Liu, Jiao Yin, Bo Feng, Tao Xu, Fu Wang

**Affiliations:** 1 Laboratory of Environmental Sciences and Technology, Xinjiang Technical Institute of Physics & Chemistry, and Key Laboratory of Functional Materials and Devices for Special Environments, Chinese Academy of Sciences, Urumqi 830011, China; liujing@ms.xjb.ac.cn (J.L.); yinjiao@ms.xjb.ac.cn (J.Y.); fengb1008@163.com (B.F.); 2 University of Chinese Academy of Sciences, No. 19 Yuquan Road, Beijing 100049, China; 3 Department of Chemistry and Biochemistry, Northern Illinois University, DeKalb, IL 60115, USA

**Keywords:** ORR, unprotected, Pt nanoclusters, carbon nanotubes, electrocatalysis

## Abstract

The Pt particles within diameters of 1–3 nm known as Pt nanoclusters (NCs) are widely considered to be satisfactory oxygen reduction reaction (ORR) catalysts due to higher electrocatalytic performance and cost effectiveness. However, the utilization of such smaller Pt NCs is always limited by the synthesis strategies, stability and methanol tolerance of Pt. Herein, unprotected Pt NCs (~2.2 nm) dispersed on carbon nanotubes (CNTs) were prepared via a modified top-down approach using liquid Li as a solvent to break down the bulk Pt. Compared with the commercial Pt/C, the resultant Pt NCs/CNTs catalyst (Pt loading: 10 wt.%) exhibited more desirable ORR catalytic performance in 0.1 M HClO_4_. The specific activity (SA) and mass activity (MA) at 0.9 V for ORR over Pt NCs/CNTs were 2.5 and 3.2 times higher than those over the commercial Pt/C (Pt loading: 20 wt.%). Meanwhile, the Pt NCs/CNTs catalyst demonstrated more satisfactory stability and methanol tolerance. Compared with the obvious loss (~69%) of commercial Pt/C, only a slight current decrease (~10%) was observed for Pt NCs/CNTs after the chronoamperometric measurement for 2 × 10^4^ s. Hence, the as-prepared Pt NCs/CNTs material displays great potential as a practical ORR catalyst.

## 1. Introduction

The oxygen reduction reaction (ORR) is a vital component in fuel cells. Currently, Pt-based nanomaterials are the most effective catalysts to improve the ORR performance [1]. However, they suffer from a series of drawbacks including high costs, unsatisfactory catalytic activity, poor stability and lower methanol tolerance [2,3]. Hence, a large number of research articles have been focusing on figuring out these problems [4,5,6]. Among a wide variety of strategies, reducing the size of Pt into smaller size less than 3 nm referred as nanoclusters (NCs) has been frequently highlighted in that Pt NCs present more satisfactory ORR performance compared with larger Pt nanoparticles (NPs) because of the smaller size, narrower size distribution and more active sites [7,8,9]. More importantly, the smaller size endows Pt NCs with a large proportion of low-coordinated surface atoms, which is helpful for the adsorption and activation of O_2_ on the catalyst surface [10]. As a consequence, Pt NC catalysts adopted in ORR can both dramatically enhance the electrocatalytic activity and lower the cost of Pt. However, Pt NCs usually require to be protected by the stabilizing agents to prevent the aggregation, which would hinder the mass transfer process and electron transportation and thus give rise to a sharp reduction in the electrochemical performance [11]. Consequently, developing more versatile preparation methods without any stabilizing agents to promote unprotected Pt NCs with improved electrocatalytic performance in ORR is highly desirable.

Among various fabrication strategies for the preparation of unprotected Pt NCs, the top-down approach is regarded as one of the most effective methods to relatively regulate the uniform distribution of particle size and control the particle size within 1–3 nm [12]. In particular, this method not only involves no protecting agents but also avoids chloride contamination which will also likely cause the loss of catalytic activity as protecting agents [13,14]. For example, Vulcan XC-72R carbon black supported Pt (Pt/Vulcan XC-72R) with a mean particle size of 1.9 ± 0.3 nm was synthesized via this method. Though the Pt/Vulcan XC-72R exhibited improved ORR activity compared with commercial Pt/C, it is far from to meet the requirement for practical application of fuel cells. In addition, the stability of the Pt/Vulcan XC-72R is unsatisfactory and the Pt loading on the substrate of Vulcan XC-72R was up to 15 wt.%. Hence, how to continue to lower the mass loading and boost the electrocatalytic performance and stability of unprotected Pt NCs stemmed from the top-down approach are urgently needed to be addressed.

Furthermore, the substrates to uniformly load and anchor the unprotected Pt NCs also make great contributions to promoting catalytic activity and stability for ORR. In particular, the strong interactions between the support and Pt NCs can prevent the dissolution and aggregation of Pt NCs during the ORR process [15]. Among a wide variety of available supporting materials, CNTs have been proposed as one of the most promising matrixes because of the high resistance to electrochemical oxidation and corrosion, high electronic conductivity, and low cost [16,17,18]. More importantly, the graphitic structure endows CNTs with higher electrochemical stability and quicker electron transfer rate, which is beneficial for both improving the ORR performance and stability [19,20]. Hence, CNTs as supporting material would give rise to an enhanced ORR activity and stability.

In the present work, a modified top-down method was proposed to fabricate unprotected Pt NCs anchored on CNTs with the average particle size of ~2.2 nm. The as-prepared Pt NCs/CNTs-10% demonstrated more desirable ORR activity in 0.1 M HClO_4_. The specific activity (SA) and mass activity (MA) over Pt NCs/CNTs-10% at 0.9 V were calculated to be 0.194 mA cm^−2^ and 167 mA mg^−1^, respectively, which were 2.5 times and 3.2 times higher than those of Pt/C-20% (0.079 mA cm^−2^ and 52 mA mg^−1^). Notably, such a sample also exhibited remarkably higher stability and methanol tolerance than commercial Pt/C-20%. Compared with the obvious loss (~69%) of commercial Pt/C, only a slight current decrease (~10%) was observed after the chronoamperometric measurement for 2 × 10^4^ s. Thus, the as-prepared Pt NCs/CNTs-10% holds great potential as a practical ORR catalyst.

## 2. Materials and Methods 

### 2.1. Materials

Commercial Pt/C catalyst (Pt loading: 20 wt.%) and carbon nanotubes were obtained from Adamas-beta (Shanghai, China). Lithium and platinum foil, perchloric acid (70%) were purchased from Alfa-Aesar (Shanghai, China). Absolute ethyl alcohol and methanol were supplied by Tianjin Zhi yuan Chemical Reagent Co., Ltd (Tianjin, China). Nafion (5%) were purchased from Sigma-aldrich (Saint Louis, MO, USA).

### 2.2. Synthesis of Pt NCs/CNTs-10% 

Synthesis of Pt NCs was carried out through a modified top-down approach in an Ar-filled glovebox. 2.1 g of Li was heated to 235 °C in a nickel crucible, to which 20 mg Pt foil was added simultaneously. The mixture was sonicated at 235 °C for 5 h to achieve a homogeneous dispersion. Then the Li-Pt molten was poured onto a stainless plate and removed from the glovebox. By cutting into small pieces, Li was slowly converted to LiOH under vapor water. 180 mg of CNTs was added into the LiOH. The mixture was pestled for 30 min. The LiOH was isolated with distilled water leaving the Pt NCs on the CNTs support (Pt NCs/CNTs). The Pt NCs/CNTs was washed five times with distilled water and then dried at 60 °C in vacuum for 12 h. The sample with 10 wt.% Pt loading and the commercial Pt/C with 20 wt.% Pt loading are denoted as Pt NCs/CNTs-10% and Pt/C-20%, respectively.

### 2.3. Characterizations

X-ray diffraction (XRD) patterns were performed with samples on a Bruker D8 diffractometer, (Karlsruhe, Germany). Transmission electron microscope (TEM) images were obtained with a FEI f20. The lattice-resolved images were obtained by high-resolution TEM (HRTEM, FEI f20, Hillsboro, OR, USA). The Energy dispersive X-ray spectroscopy (EDS) was obtained by the EDS8000. X-ray photoelectron spectroscopy (XPS) was obtained on a KRATOS Analytical AXISHSi spectrometer (Kanagawa, Japan). N_2_ adsorption/desorption isotherms were made on Micromeritics ASAP2020 (Norcross, GA, USA). The inductively coupled plasma-optical emission spectrometry (ICP-OES) data were obtained on VISTA-PRO CCD (Chicago, IL, USA).

### 2.4. Electrocatalytic Measurements

Cyclic voltammograms (CV) and rotating disk electrode (RDE, Pine Instrument, Hong Kong, China) measurements were conducted on CHI660E to evaluate the ORR performance. All data were recorded at room temperature in a three-electrode cell in 0.1 M HClO_4_. Pt foil and Ag/AgCl electrodes are used as counter electrode and reference electrode, respectively. All potentials in this work are reported versus reversible hydrogen electrode (RHE) via converting the measured potentials using Equation (1):

(1) $$\;E_{RHE}=E_{Ag/AgCl\;}+0.059\times pH+0.197\;$$

Prior to the CV and RDE measurements, the glassy carbon electrode was polished with 0.3 μm alumina slurries (Buehler, IL, USA) to a mirror finish and then washed and ultrasonically cleaned in ultrapure water for 3 min. The 0.1 M HClO_4_ electrolyte was saturated with O_2_ (99.999%), N_2_ (99.999%) or Ar (99.999%). The working electrode was prepared as follows: Suspend 6 mg Pt NCs/CNTs-10% in the mixture of Nafion (25 μL, 5 wt.%) and C_2_H_5_OH (475 µL) and then sonicated for 30 min to obtain a homogeneous suspension. 10 μL of the suspension was deposited on the Φ5 mm glassy carbon disk and dried in air. The Pt loading on the glassy carbon was ∼0.06 mg cm^−2^. CV data were measured between 0 and 1.2 V at 50 mV s^−1^. RDE data were obtained at 10 mV s^–1^. The stability and the methanol tolerance of Pt NCs/CNTs-10% were investigated by the chronoamperometric measurement.

## 3. Results and Discussion

### 3.1. Physicochemical Characterizations

**Figure 1:**
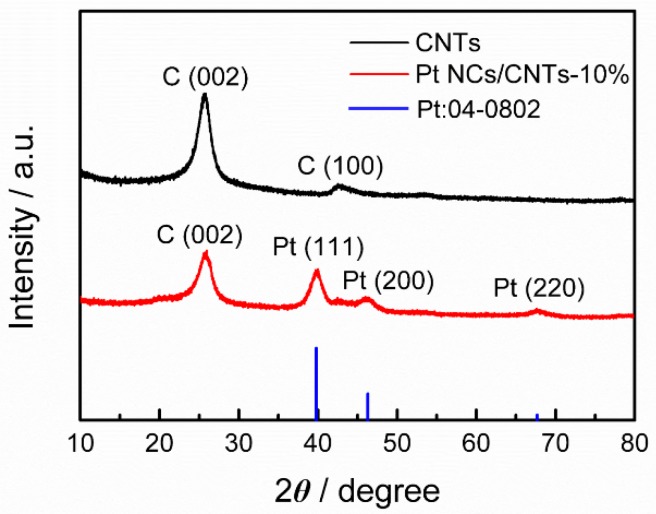
X-ray diffraction (XRD) patterns of the carbon nanotubes (CNTs), Pt NCs/CNTs-10%, and standard bulk Pt.

XRD patterns are conducted to characterize the crystal structure of Pt NCs/CNTs-10%. As depicted in Figure 1, a typical fcc crystal phase is found for Pt NCs/CNTs-10%. The as-prepared Pt NCs/CNTs-10% exhibits the same diffraction patterns as the standard Pt card (JCPDS: 04-0802). The XRD pattern of CNT support displays two peaks at ~25.7° and ~42.5° assigned to the (002) and (100) reflections, respectively [21]. The (002) peak is also clearly observed in the XRD pattern of Pt NCs/CNTs-10%. The (100) peak is weak and overlapped by the diffraction at ~46.2° attributed to the Pt (200) reflection. The mean particle size of Pt NCs is calculated to be 2.4 nm from the Pt (111) diffraction using the Scherrer equation. The geometric surface area of Pt NCs is determined to be 116.8 m^2^ g^−1^ from the XRD analysis using the Equation (2):

(2) $$\;s=\frac{6000}{\rho d\;}\;$$

where *ρ* is the density of Pt (21.4 g cm^–3^) and d/nm is the average particle size determined by the XRD pattern.

**Figure 2:**
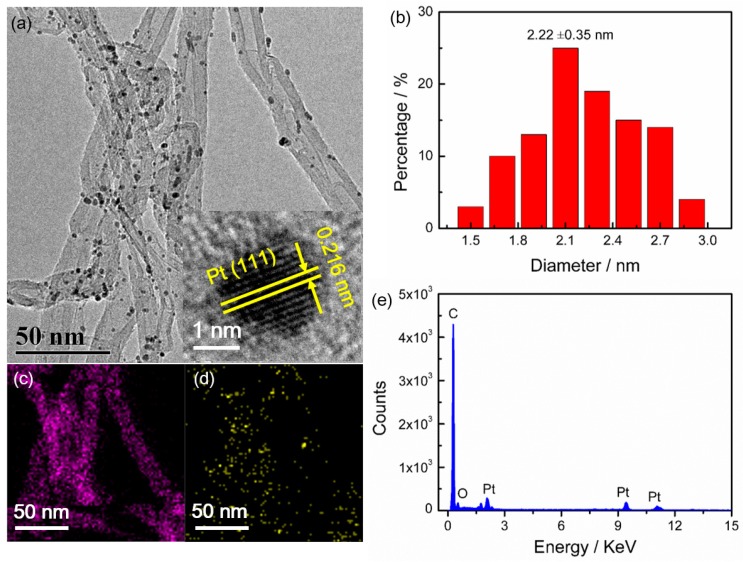
(**a**) Transmission electron microscope (TEM) image; (**b**) histograms of Pt NCs size distribution; (**c**) C mapping; (**d**) Pt mapping and (**e**) Energy dispersive X-ray spectroscopy (EDS) spectrum of Pt NCs/CNTs-10%.

**Figure 3:**
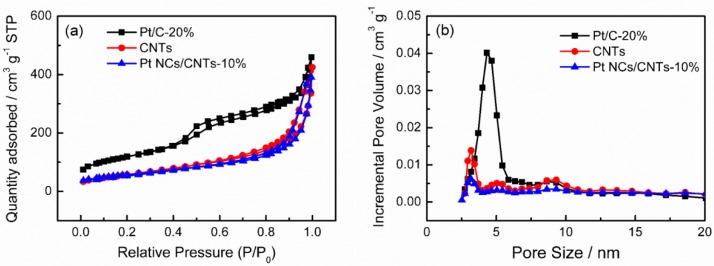
(**a**) N_2_ adsorption/desorption isotherms and (**b**) pore size distributions of CNTs, Pt NCs/CNTs-10%, and Pt/C-20%.

Figure 2a shows the representative TEM image of Pt NCs/CNTs-10%. It can be seen that the Pt NCs are uniformly dispersed on CNTs with average diameter size of 2.2 ± 0.35 nm (Figure 2b), which is consistent with the XRD result (2.4 nm). The inset of Figure 1 shows the HRTEM image of the Pt NCs. The Pt (111) surface orientation is determined through investigating the distance between two columns of Pt atoms. The corresponding elemental mapping (Figure 2c,d) and selected-area EDS (Figure 2e) analyses further clearly demonstrate the uniform distribution of Pt NCs on the CNTs. According to the ICP-OES analysis, the Pt mass loading is about 8.7%, which slightly dropped from the theoretical value of 10% probably caused by the limited surface area of CNTs, which is determined to be 253 m^2^ g^–1^ according to the N_2_ absorption/desorption isotherms (Figure 3). The Brunauer-Emmett-Teller (BET) surface area of Pt NCs/CNTs-10% and Pt/C-20% are also carried out, which are determined to be 217 m^2^ g^−1^ and 411 m^2^ g^–1^, respectively. Obviously, the BET surface area of Pt NCs/CNTs-10% is smaller than that of CNTs, which can be ascribed to the framework shrinkage during the process of preparing the catalyst.

**Figure 4:**
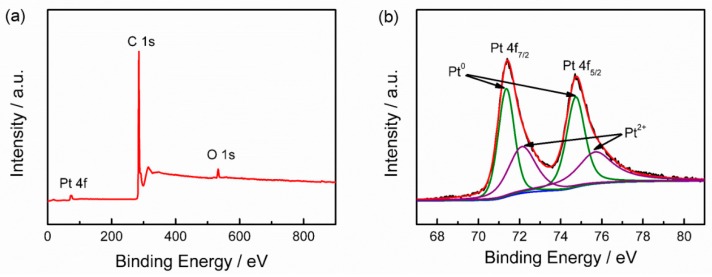
(**a**) Survey XPS spectrum and (**b**) Pt 4f spectrum of Pt NCs/CNTs-10%.

XPS measurement is employed to analyze the chemical states of Pt NCs. The C 1s peak at 284.6 eV is adopted as a reference for the correction of the binding energy positions [22]. As shown in Figure 4a, both C and Pt are detected at their specific binding energy positions. As displayed in Figure 4b, high-resolution XPS spectrum of Pt 4f presents the characteristic peaks of Pt 4f_7/2_ and Pt 4f_5/2_ at ~71.4 and ~74.7 eV, respectively, which can be divided into two pairs of doubles. The peaks appeared at ~71.35 and ~74.73 eV are characteristic for metallic Pt^0^ [23]. The peaks located at 72.12 and 75.68 eV are originated from Pt^2+^ [24]. Clearly, Pt^0^ is predominant. Based the deconvoluted XPS, it can be determined that the Pt NCs contain 69% metallic Pt and 31% Pt oxide. In addition, there is a slight shift of the Pt^0^ peak to higher binding energies, which may be attributed to the strong interactions between Pt NCs and CNTs [25], which are beneficial for improving the stability of Pt NCs/CNTs-10%.

### 3.2. Electrochemical Evaluation

**Figure 5:**
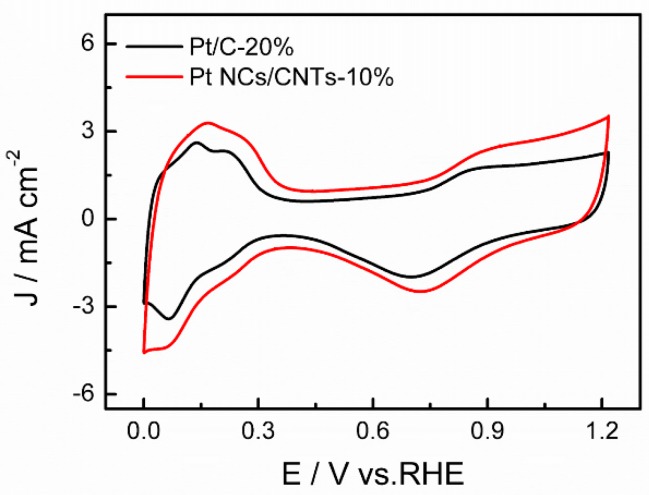
Cyclic voltammograms (CVs) of Pt NCs/CNTs-10% and Pt/C-20% in Ar-saturated 0.1 M HClO_4_ at 50 mV s^−1^.

Figure 5 shows the CVs of Pt NCs/CNTs-10% and Pt/C-20% in Ar-saturated 0.1 M HClO_4_ solution. A typical hydrogen adsorption/desorption behavior on Pt is clearly observed on both Pt NCs/CNTs-10% and Pt/C-20%. The electrochemical surface area (ECSA, m^2^ g^–1^) are calculated using Equation (3):

(3) $$\;\mathrm{ECSA}=\frac{Q_{H}}{0.21\times m_{Pt}}\;$$

where Q*_H_* (mC) is the charge due to the hydrogen adsorption in the hydrogen region of the CV. 0.21 (mC cm^–2^) is the electrical charge associated with monolayer adsorption of hydrogen on Pt. m*_Pt_* is the Pt loading on working electrode. Results indicate that the ECSA of Pt NCs/CNTs-10% and Pt/C-20% are determined to be 48.23 m^2^ g^–1^ and 38.79 m^2^ g^–1^, respectively. The higher ECSA of Pt NCs/CNTs-10% can be attributed to the small size and uniform distribution of Pt NCs on the CNTs.

**Figure 6:**
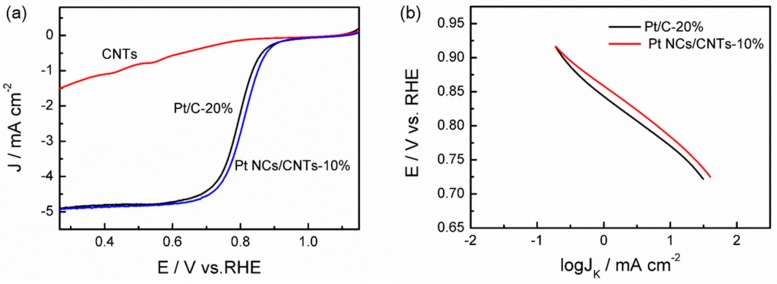
(**a**) Comparison of RDE polarization curves of Pt NCs/CNTs-10% and Pt/C-20% at 1600 rpm in O_2_-saturated 0.1 M HClO_4_ solution at 10 mV s^–1^ and (**b**) Tafel plots for ORR derived from (**a**).

The RDE measurements for ORR are carried out in O_2_-saturated 0.1 M HClO_4_ at a potential scan rate of 10 mV s^–1^. Figure 6a exhibits the liner sweep voltammetry (LSV) curves of CNTs, Pt NCs/CNTs-10% and Pt/C-20% at the rotation rate of 1600 rpm. Obviously, it can be seen that the OMC is inactive for ORR. The diffusion and diffusion-kinetic controlled regions are found for Pt NCs/CNTs-10% and Pt/C-20% in the potentials below 0.65 V and 0.65–0.95 V, respectively. It is worth mentioning that Pt NCs/CNTs-10% possess a higher onset potential (E_onset_ = 0.87 V) and half-wave potential (E_1/2_ = 0.83 V) than that of Pt/C-20% (E_onset_ = 0.86 V and E_1/2_ = 0.81 V), indicating the higher ORR activity of Pt NCs/CNTs-10%. Furthermore, the ORR diffusion limited current (J_L_) of Pt NCs/CNTs-10% is higher compared with that measured for Pt/C-20%. Figure 6b presents the tafel plots determined on the basis of the RDE data at 1600 rpm. The slope value for Pt NCs/CNTs-10% is 81 mV decade^−1^, which is smaller than that of Pt/C-20% (87 mV decade^−1^), further indicating the more efficient ORR process on Pt NCs/CNTs-10%.

**Figure 7:**
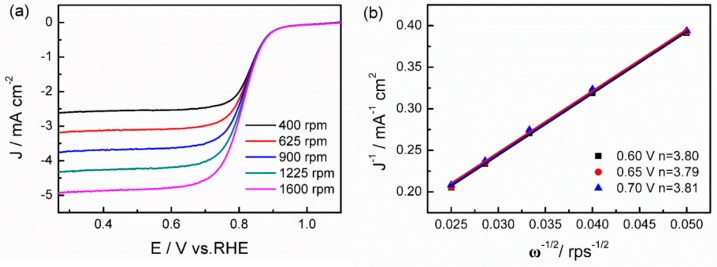
(**a**) RDE polarization curves for ORR on Pt NCs/CNTs-10% at different rotation speeds from 400 to 1600 rpm in O_2_-saturated 0.1 M HClO_4_ solution at 10 mV s^–1^ and (**b**) Corresponding K-L plot at different potentials.

The LSV curves of Pt NCs/CNTs-10% at different rotation speeds are further performed to clarify the ORR pathway. As displayed in Figure 7a, the LSV curves of Pt NCs/CNTs-10% exhibit rotating-speed-dependent current densities. Potential in the diffusion and diffusion-kinetic regions, varying from 0.6 to 0.7 V, was chosen to calculate the values of electron transfer number (n) by Koutecky-Levich (K-L) Equation (4):

(4) $$\;\frac{1}{J}=\frac{1}{J_{K}}+\frac{1}{J_{L}}=\frac{1}{J_{K}}+\frac{1}{{B\omega }^{1/2}}=\frac{1}{J_{K}}+\frac{1}{0.62{\mathrm{nFCD}}^{2/3}v^{-1/6}\omega^{1/2}}\;$$

where J, J_L_, and J_K_ is the measured current, diffusion limited current, and kinetic current, respectively. ω is the electrode rotating rate. B is determined from the slope of the K-L plots. Faraday constant (F) is 96485 C. Diffusion coefficient (D) is 1.9 × 10^–5^ cm^2^ s^–1^. Kinematic viscosity of the electrolyte (ν) is 0.01 cm^2^ s^–1^ and bulk concentration of O_2_ (C) is 1.2 × 10^–6^ mol cm^−3^. As shown in Figure 7b, the corresponding K-L plots show excellent linearity, indicating the the first-order reaction kinetics. Meanwhile, the slopes of the K-L plots are almost same, suggesting a similar values of n. Based on the K-L slopes, the value of n can be determined to be 3.80 (0.60 V), 3.79 (0.65 V), and 3.81 (0.70 V), respectively. It suggests that Pt NCs/CNTs-10% proceeds dominantly with a preferential 4e^−^ pathway, which is beneficial to improve the efficiency of fuel cells.

The SA and MA are important parameters for investigating intrinsic activities of catalysts. Here, the SA and MA of Pt NCs/CNTs-10% and Pt/C-20% are calculated from J_K_ and normalization with the Pt surface area and loading, respectively. The J_K_ is calculated from the mass-transport correction of RDE using Equation (5):

(5) $$\;J_{K}=\frac{J_{L}\times J}{J_{L}-J}\;$$

**Figure 8:**
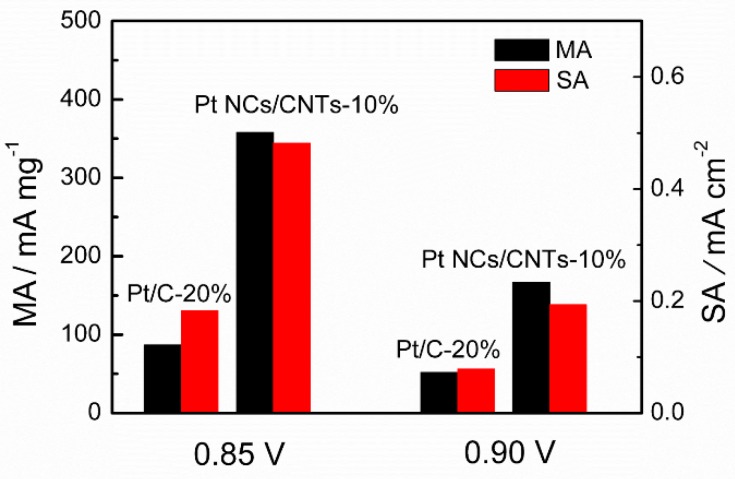
Mass activity (MA) and specific activity (SA) of Pt NCs/CNTs-10% and Pt/C-20% in 0.1 M HClO_4_ at 0.85 V and 0.9 V.

As shown in Figure 8, at 0.85 V, the SA over Pt NCs/CNTs-10% (0.482 mA cm^–2^) is 2.6 times larger than that of Pt/C-20% (0.183 mA cm^–2^) and the MA (358 mA mg^–1^) is 4.1 times higher than that of Pt/C-20% (87 mA mg^–1^ Pt). These values over Pt NCs/CNTs-10% at 0.9 V are calculated to be 0.194 mA cm^–2^ and 167 mA mg^−1^ respectively, which are 2.5 and 3.2 times higher than those of Pt/C-20% (0.079 mA cm^–2^ and 52 mA mg^–1^). The values of Pt NCs/CNTs-10% are not only superior to that of Pt/C-20%, but also that of most reported Pt-based catalysts as presented in Table 1.

The remarkable ORR activity of Pt NCs/CNTs-10% catalyst can be attributed to the three factors: First, the reduced coordination of Pt NCs as well as the decreased electrophilicity give rise to a corresponding reduction in the activation energy of the dissociative chemisorption of O_2_, facilitating the 4e^−^ transfer process [36]. Second, the unprotected Pt NCs with smaller size and narrower size distribution can provide more active sites and promote the charge transfer during the ORR process. Third, CNTs with graphitic structure exhibit higher electron transfer rate [37]. All these together make the as-prepared Pt NCs/CNTs-10% exhibit more desirable ORR performance.

### 3.3. Electrochemical Stability and Methanol Tolerance

**Figure 9:**
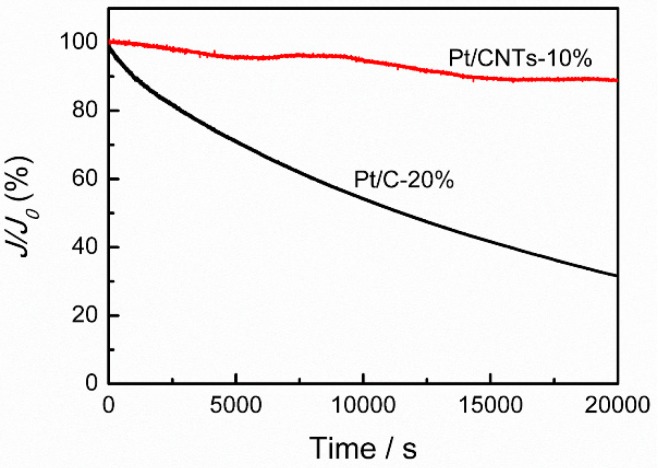
Relative current-time profiles of Pt NCs/CNTs-10% and Pt/C-20% in 0.1 M O_2_-saturated HClO_4_.

**Figure 10:**
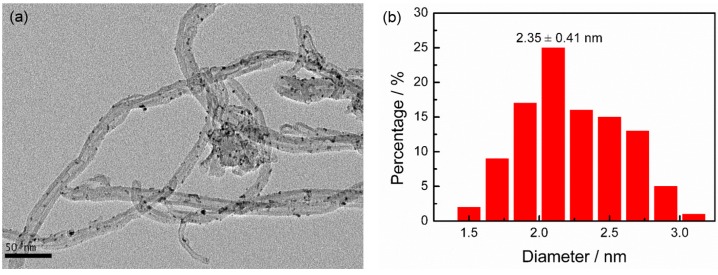
(**a**) TEM image and (**b**) size distribution of Pt NCs/CNTs-10% after chronoamperometric measurement for 2 × 10^4^ s in O_2_-saturated 0.1 M HClO_4_.

Besides the ORR activity, the stability of catalysts are also important for the practical application of fuel cells. Here, the stability of Pt NCs/CNTs-10% is performed by chronoamperometric measurement, which is an effective approach to investigate the stability of catalysts [38]. For comparison, Pt/C-20% is also tested as a baseline catalyst. As shown in Figure 9, after chronoamperometric measurement for 2 × 10^4^ s, only ~10% current decrease is observed on Pt NCs/CNTs-10%. However, the value for Pt/C-20% is ~69%, much larger than that of Pt NCs/CNTs-10%, demonstrating a more satisfactory stability of Pt NCs/CNTs-10%. As reported in literatures, the poor stability of Pt/C-20% is mainly derived from the agglomeration of Pt particles [39]. Hence, TEM image of Pt NCs/CNTs-10% after the chronoamperometric measurement is conducted. As shown in Figure 10, no obvious aggregation is observed and the average particle size is determined to be ~2.35 ± 0.41 nm. According to the aforementioned results, we can conclude that the excellent stability of Pt NCs/CNTs-10% may be attributed to the trapping the Pt NCs in the CNTs nano-network as well as the interactions between Pt NCs and CNTs, suppressing the migration, aggregation, and growth in size of the small Pt NCs. 

**Figure 11:**
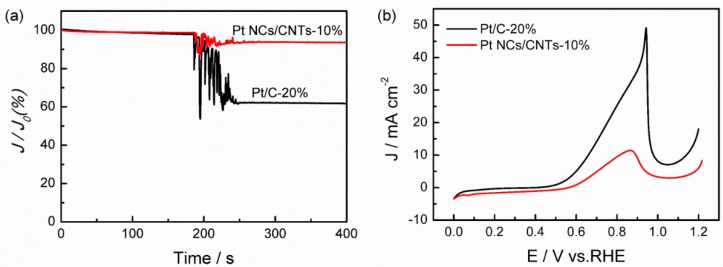
(**a**) Chronoamperometric responses of Pt NCs/CNTs-10% and Pt/C-20% in 0.1 M O_2_-saturated HClO_4_ before and after addition of methanol; (**b**) Polarization curves for methanol oxidation on Pt NCs/CNTs-10% and Pt/C-20% catalysts in 0.1 M HClO_4_ and 0.5 M CH_3_OH solution saturated with N_2_ at scan rate of 10 mV s^–1^.

The crossover of methanol from the anode to the cathode can lead to a reduction in cell voltage by ~200–300 mV. There is a competitive reaction between ORR and methanol oxidation on Pt-based cathodes. Therefore, it is highly desirable to develop ORR electrocatalysts with a high methanol tolerance for the application of fuel cells. Figure 11 displays the methanol tolerance of Pt NCs/CNTs-10% and Pt/C-20% evaluated by adding methanol into the electrolyte during the chronoamperometric measurement. It can be seen that the current of Pt/C-20% decreases to 63% after methanol injection. In contrary, only a slight oscillation in the current is observed for Pt NCs/CNTs-10%, suggesting a great methanol tolerance of Pt NCs/CNTs-10%. The methanol oxidation in 0.1 M N_2_-saturated HClO_4_ solution containing 0.5 M CH_3_OH is further performed to analyze the origin of the high methanol tolerance of Pt NCs/CNTs-10%. As depicted in Figure 11b, the methanol oxidation current densities on Pt NCs/CNTs-10% is 11.5 mA cm^–2^, which is much smaller than that of Pt/C-20% (49.4 mA cm^–2^) implying that the methanol oxidation on Pt NCs/CNTs-10% is less active than that on Pt/C-20%, which can be attributed to the hindrance of chemisorption of methanol on the catalyst surface. Thus, the high methanol tolerance of the Pt NCs/CNTs-10% can be explained by the preferential adsorption of O_2_ over methanol on the Pt NCs site.

## 4. Conclusions

In summary, highly dispersed Pt NCs (~2.2 nm) anchored on CNTs were successfully fabricated through a modified top-down strategy. The Pt NCs/CNTs-10% (Pt loading: 10 wt.%) exhibited superior catalytic activity, stability, and methanol tolerance compared with Pt/C-20%. The enhanced activity of Pt NCs/CNTs-10% is possibly ascribed to the reduced electrophilicity and the decreased coordination of the Pt NCs that lead to a decrease in the activation energy of O_2_, the smaller size and narrower size distribution of unprotected Pt NCs that provide more active sites, and the graphitic structure of CNTs that can improve the electron transfer rate. The Pt NCs/CNTs-10% would be a promising catalyst for fuel cells.

## Figures and Tables

**Table 1:** Comparison of ORR performances for the present Pt NCs/CNTs-10% and the reported Pt-based catalysts in acid solution at 0.9 V. (NA: not available).

Electroatalyst	Pt Loading	ORR Performances		References
		MA/mA mg^–1^	SA/mA cm^–2^	
Pt NCs/CNTs	8.7 wt.%	167	0.194	This work
Pt_1_Pd_3_/OMC	18.96 wt.%	14.02	0.034	[26]
Pt_3_Co/C	18.1 wt.%	321	0.501	[27]
PtCo/C	20 wt.%	9.9	0.016	[28]
Pd_80_Cu_6_Pt_14_	23.5 wt.%	420	0.5	[29]
Pt-Vulcan	NA	117	0.19	[30]
Pt-Cr(1:1)/C	10 wt.%	65	0.175	[31]
Pt nanocubes	100 wt.%	NA	0.113	[32]
Pt/GMC	22 wt.%	140	0.208	[33]
Pt/MWCNT	NA	16.9	0.23	[34]
Pt_0.65_Ni_0.35_/C	17.2 wt.%	90	0.15	[35]
